# Protein complexes from mouse and chick brain that interact with phospho-KXGS motif tau/microtubule associated protein antibody

**DOI:** 10.1242/bio.060067

**Published:** 2024-02-27

**Authors:** D. S. Davies, A. T. Arthur, H. L. Aitken, B. Crossett, C. S. Goldsbury

**Affiliations:** ^1^ Faculty of Medicine and Health, School of Medical Sciences, Brain and Mind Centre, The University of Sydney, Sydney, NSW 2050, Australia; ^2^ Sydney Mass Spectrometry, The University of Sydney, Sydney, NSW 2050, Australia

**Keywords:** Tau, Microtubule associated protein 2, MAP2, KXGS motif, Cofilin, Actin depolymerizing factor, ADF, Eukaryotic translation initiation factor 3, Eif3, FSD1l

## Abstract

Mouse monoclonal 12E8 antibody, which recognises conserved serine phosphorylated KXGS motifs in the microtubule binding domains of tau/tau-like microtubule associated proteins (MAPs), shows elevated binding in brain during normal embryonic development (mammals and birds) and at the early stages of human Alzheimer’s disease (AD). It also labels ADF/cofilin-actin rods that form in neurites during exposure to stressors. We aimed to identify direct and indirect 12E8 binding proteins in postnatal mouse brain and embryonic chick brain by immunoprecipitation (IP), mass spectrometry and immunofluorescence. Tau and/or MAP2 were major direct 12E8-binding proteins detected in all IPs, and actin and/or tubulin were co-immunoprecipitated in most samples. Additional proteins were different in mouse versus chick brain IP. In mouse brain IPs, FSD1l and intermediate filament proteins – vimentin, α-internexin, neurofilament polypeptides – were prominent. Immunofluorescence and immunoblot using recombinant intermediate filament subunits, suggests an indirect interaction of these proteins with the 12E8 antibody. In chick brain IPs, subunits of eukaryotic translation initiation factor 3 (EIF3) were found, but no direct interaction between 12E8 and recombinant Eif3e protein was detected. Fluorescence microscopy in primary cultured chick neurons showed evidence of co-localisation of Eif3e and tubulin labelling, consistent with previous data demonstrating cytoskeletal organisation of the translation apparatus. Neither total tau or MAP2 immunolabelling accumulated at ADF/cofilin-actin rods generated in primary cultured chick neurons, and we were unable to narrow down the major antigen recognised by 12E8 antibody on ADF/cofilin-actin rods.

## Teaser

**Summary:** Immunoprecipitation (IP), mass spectrometry and immunofluorescence were used to identify potential direct and indirect binding partners of phospho-KXGS motif tau/microtubule associated protein antibody.

## INTRODUCTION

Microtubule associated proteins (MAPs) tau and MAP2 are distributed in neuronal axons and dendrites, respectively, where they regulate microtubule dynamics and have other functions (Brandt et al., 2020; Wang and Mandelkow, 2016). They belong to a family of MAPS with conserved serine phosphorylation KXGS motifs within tandem microtubule binding domains (MTBD). Phosphorylation at these motifs dynamically favours free tubulin over microtubule assembly (Drewes et al., 1995). At least 45 serine, threonine and tyrosine sites along the tau polypeptide can be phosphorylated, and many of these sites, including KXGS motifs, have increased phosphorylation during embryonic development compared to adult tau (Seubert et al., 1995; Wang and Mandelkow, 2016; Yoshida and Goedert, 2002). Increased tau phosphorylation at multiple sites also occurs in neurons in Alzheimer's disease (AD), accompanied by mis-localisation of tau from axons to the somatodendritic domain (Wang and Mandelkow, 2016). Polypeptide site-specific patterns of tau phosphorylation occur differentially with increasing disease severity, and one of the earliest sites to be hyperphosphorylated in AD are the MTBD KXGS motifs correlating with the first signs of tau mislocalisation and neurofibrillary aggregation (Augustinack et al., 2002).

There is a great degree of homology in MTBD tandem repeat protein sequences in MAP2/tau-like proteins from humans to rodents, birds and other species, as well as shared developmental patterns of phosphorylation, that indicate important broadly conserved fundamental physiological functions of these MAPs (Yoshida and Goedert, 2002). The binding partners and functionalities of the different phosphorylation sites are not yet fully understood (Wang and Mandelkow, 2016). The mouse monoclonal ‘12E8’ antibody recognises phosphorylated KXGS motifs in the MTBD of tau and MAP2 and it also labels actin-rich growth cone tips of neuronal processes, actin-depolymerising factor (ADF)/cofilin-actin rods in cultured neurons, and neuritic inclusions relating to tau toxicity in *Drosophila* (Biernat et al., 2002; Nishimura et al., 2004; Seubert et al., 1995; Whiteman et al., 2009). Phosphorylation of KXGS motifs appears to promote the binding of tau or MAP2 to cytoplasmic actin (Biernat et al., 2002; Ozer and Halpain, 2000). Better characterisation of the repertoire of binding partners of 12E8 is needed to more clearly interpret research findings that have used this antibody to evaluate cellular functions of tau/tau-like proteins and their hyperphosphorylation at KXGS motifs in neurodegenerative disease models. We performed immunoprecipitation (IP) to identify direct and indirect binding partners of this antibody in neonatal mouse and embryonic chick brain tissue to better understand its reactivity and significance. Tau, MAP2 as well as other interacting protein complexes were identified that support possible functions for MAPs beyond the stabilisation of microtubules, including interaction with the RNA translation apparatus.

## RESULTS

### The 12E8 antibody recognises epitopes on tau as well as other proteins

**Fig. 1.**
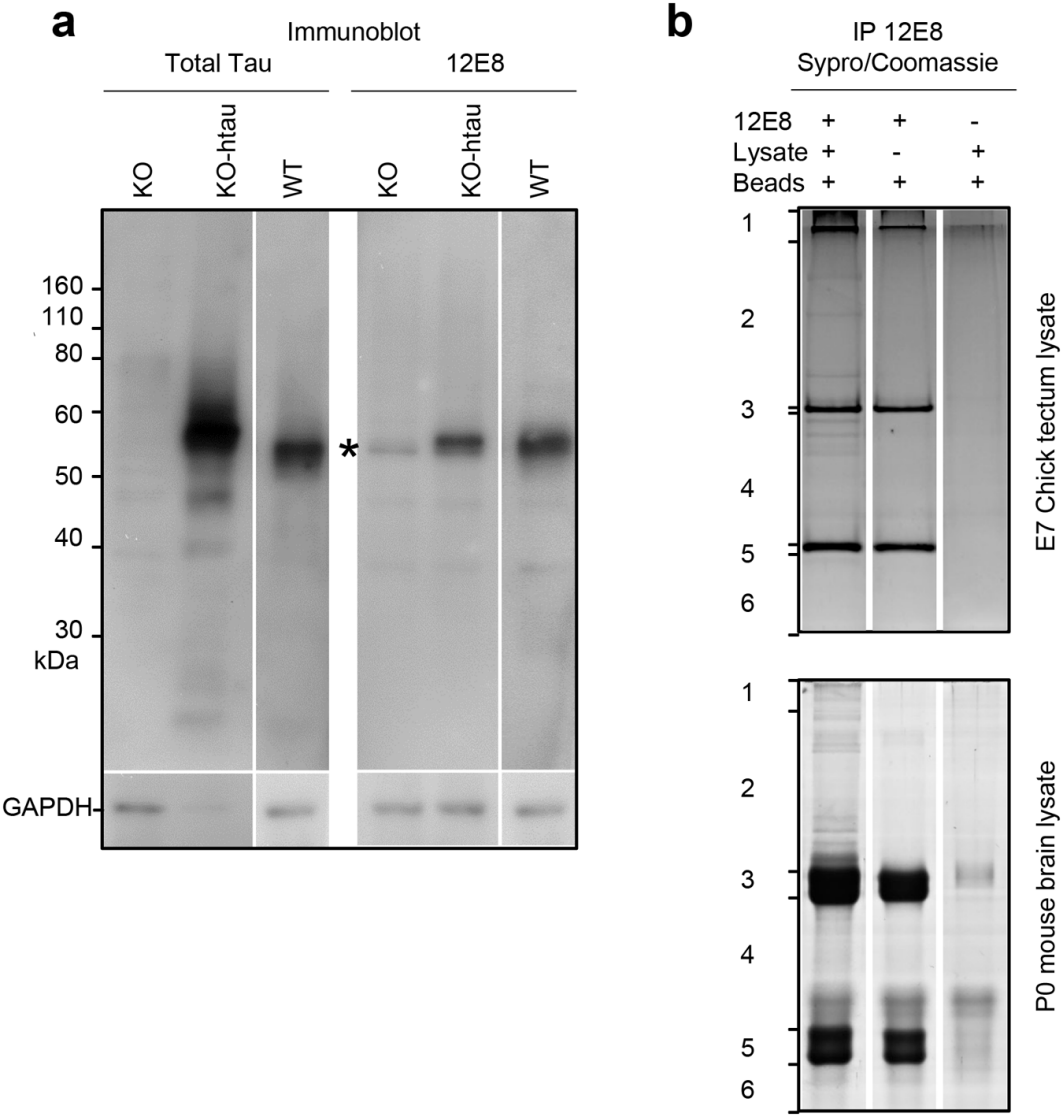
**12E8 antibodies immunoprecipitation using mouse and chick brain lysates.** (A) 12E8 binds tau protein and non-tau antigens on immunoblots from mouse brain lysates. Lysates were mouse tau null (KO), mouse tau null-human tau transgenic (KO-htau) and wild-type (WT) P0 mouse brain. Although most of the 12E8 reactivity was tau-related, as evidenced by significant reduction of band intensities in the KO compared to KO-htau or WT, a prominent∼50 kDa non-tau band reactive with 12E8 is evident all brain lysates including the KO (asterisk). (B) Sybro stained SDS gels of 12E8 mouse and chick brain immunoprecipitates. Brain lysates were used for IP with the 12E8 antibody. The resulting immunoprecipitates and experimental controls were run on 1D 8% SDS PAGE gels, stained with Sypro Ruby and counter stained with Coomassie Blue. Regions of the gel (marked 1-6 on the left) were cut out, protein extracted, and tryptic digests analysed by mass spectrometry. Regions 3 and 5 contained the Ig heavy and light chains.

Investigating the reactivity of the phospho-MAP2/tau antibody 12E8 is important because this reagent marks the earliest initiating pathological changes occurring in AD that relate to functional dysregulation of tau protein (Augustinack et al., 2002), and it is used extensively in research involving cellular and animal models of disease (Cook et al., 2014; Nishimura et al., 2004; Petry et al., 2017; Zempel et al., 2013). Reactivity of 12E8 antibody against tau protein is evident in immunoblots from mouse brain lysates, where increased reactivity is seen in human tau (htau) transgenic, relative to wild-type mouse brain, and diminished, but not zero, reactivity is evident in tau null mouse brain (Fig. 1A). Immunoblots of tau null mouse brain showed no bands for tau using total tau antibodies (Dako), but a weak band of 50-60 kDa is still seen using the 12E8 antibody (Fig. 1A) (the same observation was reported by Petry et al., 2017). Therefore, at least some of the 12E8 antibody reactivity in mouse is not tau.

### Tau, MAP2, and other proteins are immunoprecipitated by the 12E8 antibody from mouse or chick brain lysates

To further evaluate the reactivity of the 12E8 antibody, mouse (wild type, tau null and tau null-htau transgenic) and chick brain lysates were used to immunoprecipitate and identify 12E8 antibody binding antigens using mass spectrometry. Immunoprecipitated eluates were separated from Ig heavy and light chains by gel electrophoresis (Fig. 1B), then tryptic digests of proteins excised from gel sections were analysed by mass spectrometry. The lysates for IPs were prepared in RIPA buffer containing strong detergents that decrease protein-protein interactions, disrupting weak protein complexes. Nevertheless, as well as direct 12E8 binding antigens (tau and MAP2), we identified several likely indirect binding partners of the 12E8 antibody co-immunoprecipitated in these experiments. Table 1 summarises the detected proteins common and unique to each sample; Table 2 details the full list of proteins in each sample and Table S1 (see Supplementary Information) details the unique tryptic peptides detected by mass spectrometry from each sample.

**Table 1.**
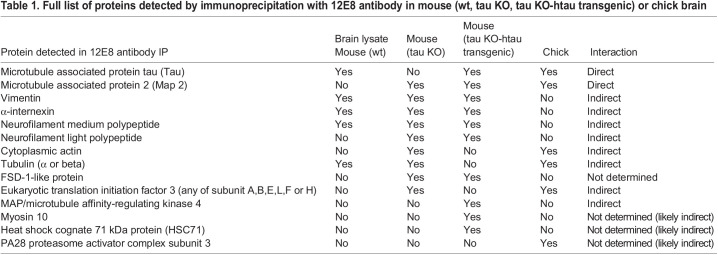
Full list of proteins detected by immunoprecipitation with 12E8 antibody in mouse (wt, tau KO, tau KO-htau transgenic) or chick brain

**Table 2.**
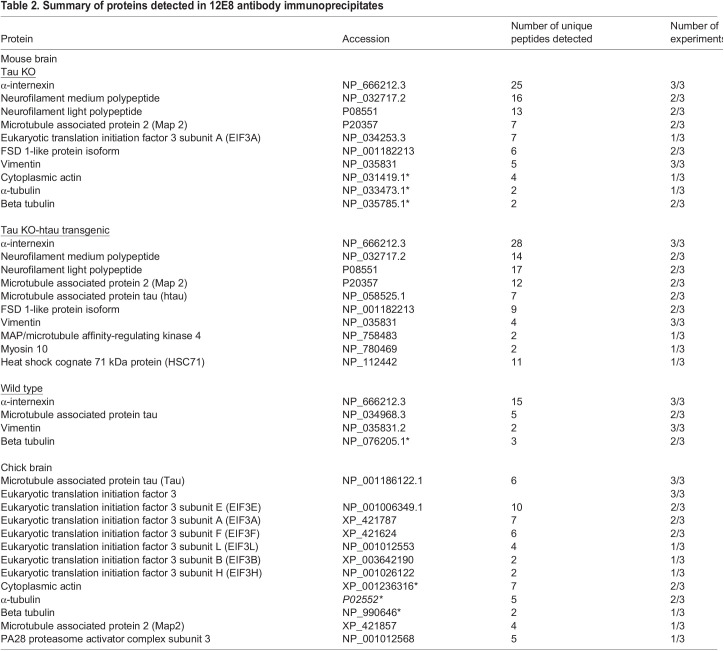
Summary of proteins detected in 12E8 antibody immunoprecipitates

Known 12E8 antigens, tau and MAP2, were identified in IP eluates from both mouse and chick brains (MAP2, and not tau was detected from tau null mice). Actin and tubulin, both known binding partners of tau and MAP2 (Fulga et al., 2007; Ozer and Halpain, 2000) were identified in IPs from mouse brain as well as chick brain. The tau/MAP2 kinase MARK4 (microtubule affinity regulating kinase) was identified in IP from htau transgenic mouse brain (this kinase is responsible for phosphorylating the specific MTBD KXGS motifs that generate the 12E8 epitope on tau and MAP2; Drewes et al., 1995; Seubert et al., 1995). FSD1l protein (Carim-Todd et al., 2001; Manabe et al., 2002; Stein et al., 2002) was in 12E8 IPs from mouse but not chick brain. Neural intermediate filament polypeptides of α-internexin and neurofilament, as well as vimentin, were also prominent in the IPs from mouse brain, but not from chick brain. In three out of three IPs from chick brains, we found subunits of the eukaryotic translation initiation factor 3 (EIF3) complex – most prevalent were subunits e or a. EIF3 complex proteins were not found in the 12E8 IPs from wild-type or htau transgenic mouse brain; however, EIF3 subunit (a) was found in one of the 12E8 IPs from tau null mouse brain.

### EIF3 subunit proteins are co-immunoprecipitated from chick brain by 12E8 antibodies in a complex with tubulin and microtubule associated protein/s

**Fig. 2.**
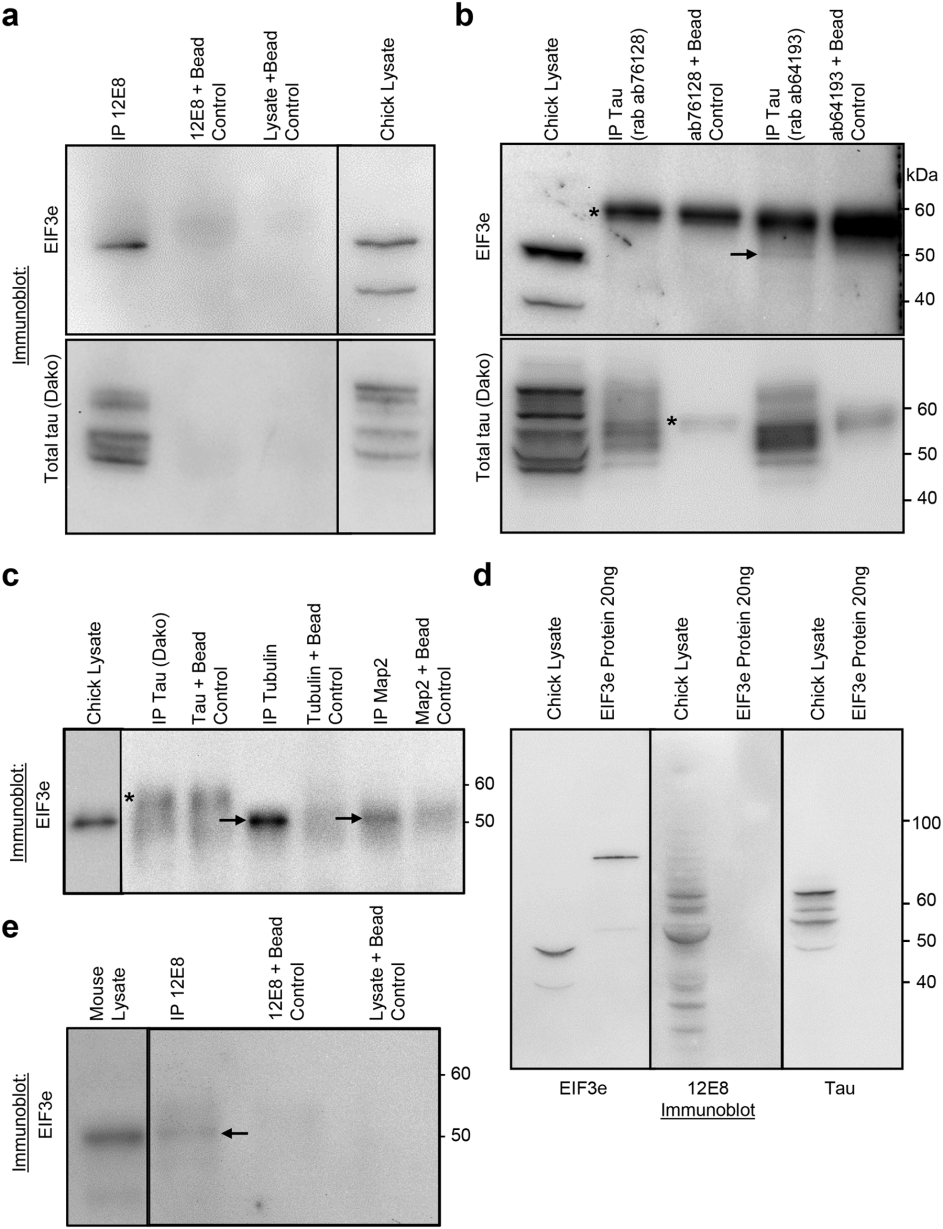
**The 12E8 antibody immunoprecipitates a complex containing MAP2, tubulin and Eif3e proteins.** (A) 12E8 antibody immunoprecipitated EIF3e and different tau isoforms from chick brain represented by at least five protein bands detected with rabbit polyclonal tau antibody (Dako) (bottom). (B) Monoclonal rabbit anti-tau antibody ab64193 weakly immunoprecipitated EIF3e (arrow) as well as several isoforms of tau from chick brain. Monoclonal rabbit anti-tau antibody ab76128 immunoprecipitated isoforms of tau but not EIF3e from chick brain. Asterisks denote the positions of rabbit Ig heavy chains. (C) Mouse monoclonal anti-α-tubulin antibodies strongly, and mouse monoclonal anti-Map2 antibodies weakly immunoprecipitated EIF3e from chick brain lysates (arrows). The asterisks denote the positions of rabbit Ig heavy chains. (D) Recombinant EIF3e protein is not directly detected by 12E8 on immunoblot. (E) 12E8 antibody weakly immunoprecipitated EIF3e from mouse brain.

As EIF3 subunit proteins were prominent in the 12E8 IPs from chick brain lysates, and one subunit (EIF3a) was in the IP from tau null mouse brain, we investigated this further. IPs were made from chick brain lysates using 12E8 antibody, MAP2 antibody, different tau antibodies or tubulin antibodies, and the presence of EIF3 subunit e (EIF3e) in the eluate determined by immunoblot (Fig. 2). This confirmed that the 12E8 antibody immunoprecipitated EIF3e along with several isoforms of tau from chick brain (Fig. 2A). MAP2 and tubulin antibodies also pulled down EIF3e from chick brain lysates (Fig. 2B). Rabbit polyclonal total tau antibody did not pull down EIF3e from chick brain lysates, but one rabbit monoclonal tau antibody (ab64193: also raised against tau MTBD KXGS epitopes) showed evidence of a very weak co-IP of EIF3e in two out of three experiments (Fig. 2C). A different rabbit monoclonal tau antibody (ab76128) did not IP EIF3e (Fig. 2C).

The EIF3e protein (48 kDa), is close in size to bands (45-70 kDa) recognised in chick and mouse brain lysate immunoblots by the 12E8 antibody (Fig. 2, Fig. 1A). However, the 12E8 antibody did not directly bind recombinant EIF3e protein on immunoblot (Fig. 2D). No 12E8 antibody reactive bands corresponding to the size of EIF3a protein (167 kDa) were detected on 12E8 immunoblots from chick or mouse brain lysates. Further, the 12E8 antibody weakly immunoprecipitated EIF3e from mouse brain (Fig. 2E), in line with the mass spectrometry result. The results suggest that the presence of EIF3 complex subunits in the 12E8 antibody IPs could be due to the direct or indirect interaction of EIF3e protein with tubulin, where due to tubulin's tight interaction with (microtubule associated) proteins that directly associate with 12E8 (MAP2 or another protein), tubulin is co-immunoprecipitated by the 12E8 antibody along with EIF3e and other complexed proteins.

### EIF3e co-localises with tubulin in primary cultured chick neurons

**Fig. 3.**
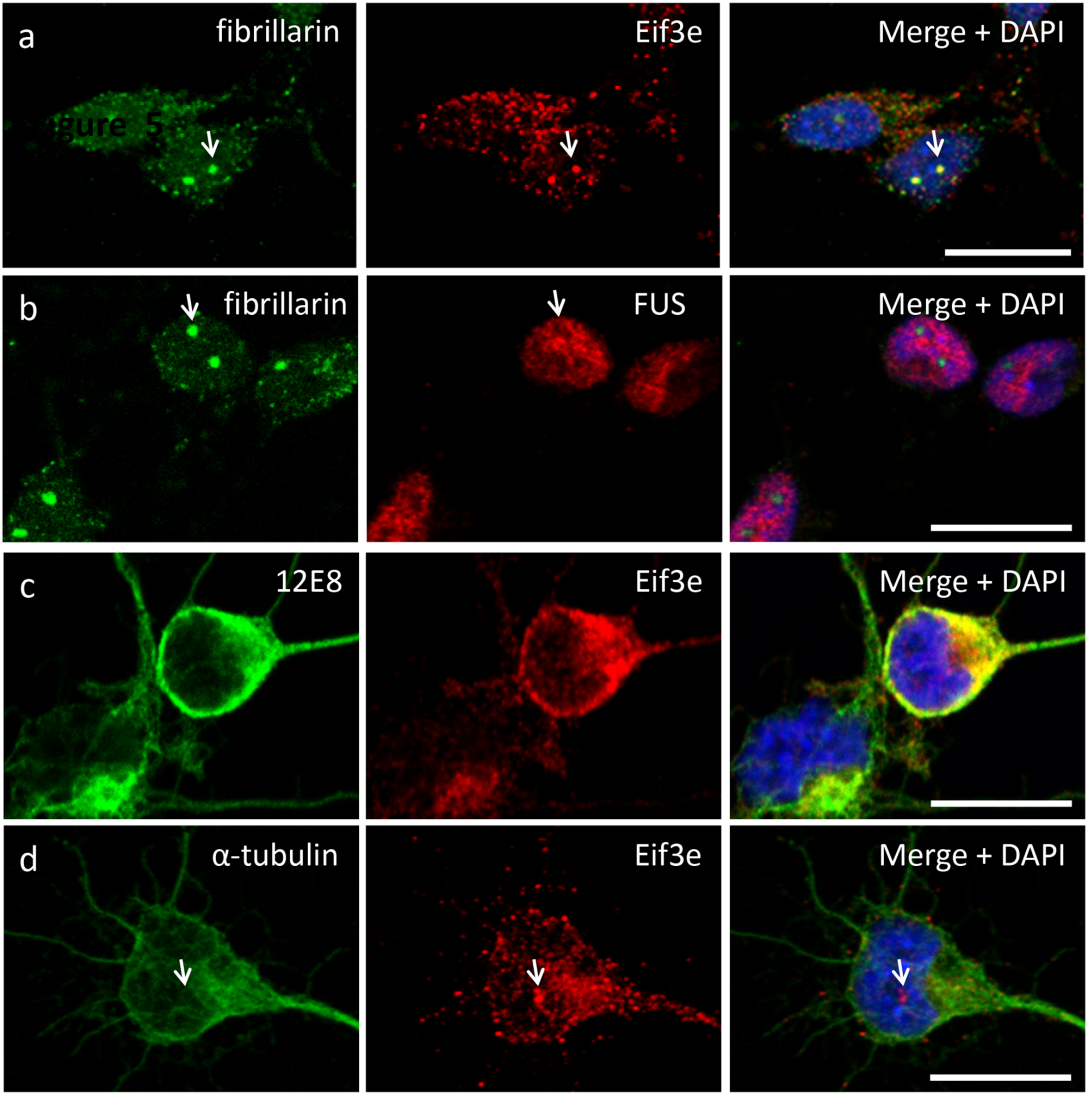
**Relative immunofluorescence localisation of 12E8, Eif3e and tubulin labelling in primary cultured chick neurons.** Immunofluorescent labelling of primary cultured chick neurons. (A) EIF3e label is prominent in the nucleolus as shown by co-localisation with nucleolar protein fibrillarin labelling (arrow). EIF3e labelling also shows punctuate distribution throughout the nucleus and cytoplasm. (B) By contrast, FUS protein labelling does not co-localise with fibrillarin label in the nucleolus. (C) The 12E8 antibody label is not evident in the nucleolus, but like EIF3e labelling, it shows a punctuate appearance in the nucleus and cytoplasm. Co-localisation analysis Pearson's and Mander's coefficients of ∼0.8 (see text) suggest some degree of co-localisation between 12E8 and EIF3e labelling. (D) EIF3e labelling shows punctuate distribution associated with tubulin or microtubules in the cytoplasm. Arrows point to nucleolus. Scale bars: 10 µm.

To evaluate the relative cellular distribution of 12E8-labelled and related proteins, primary cultured chick neurons were imaged using immunofluorescence. EIF3e antibody labelled puncta throughout the cytoplasm, and in the nucleus where labelling was concentrated in the nucleolus. Nucleolar localisation of EIF3e was confirmed by co-localisation with nucleolar marker fibrillarin (Fig. 3A). Labelling for nuclear protein FUS was excluded from the nucleolus, demonstrating the specificity of the EIF33e localisation here (Fig. 3B). The 12E8 (Fig. 3C) and tubulin (Fig. 3D) antibody labelling was evident throughout the cytoplasm and processes of neurons and lower or absent in the nucleus. The extent of ‘co-localisation’ of 12E8 and EIF3e immunolabel in the cytoplasm was high – co-localisation analysis revealed a Pearson's coefficient of 0.79 (±0.10) and Mander's coefficients of 0.76 (±0.09) and 0.78 (±0.14); *n*=6 cells (± standard deviation). These measurements do not imply direct protein-protein interactions but are an indication that EIF3e and 12E8-labelled protein/s are in close by cellular compartments. The 12E8 and EIF3e labels were not as co-localised in the nucleus – Pearson's coefficient 0.52 (± 0.22); Mander's coefficients 0.62 (±0.22) and 0.26 (±0.11), *n*=6 (± standard deviation). The 12E8 label was excluded from the nucleolus. Tubulin and EIF3e labels were also highly co-localised in the cytoplasm [Pearson's coefficient 0.75 (±0.10); Mander's coefficients 0.56 (± 0.31) and 0.82 (±0.10)], but not in the nucleus (Pearson's and Mander's coefficients≤0.1).

### Intermediate filament proteins immunoprecipitated by 12E8 do not co-localise in immunofluorescent-labelled tissue

**Fig. 4.**
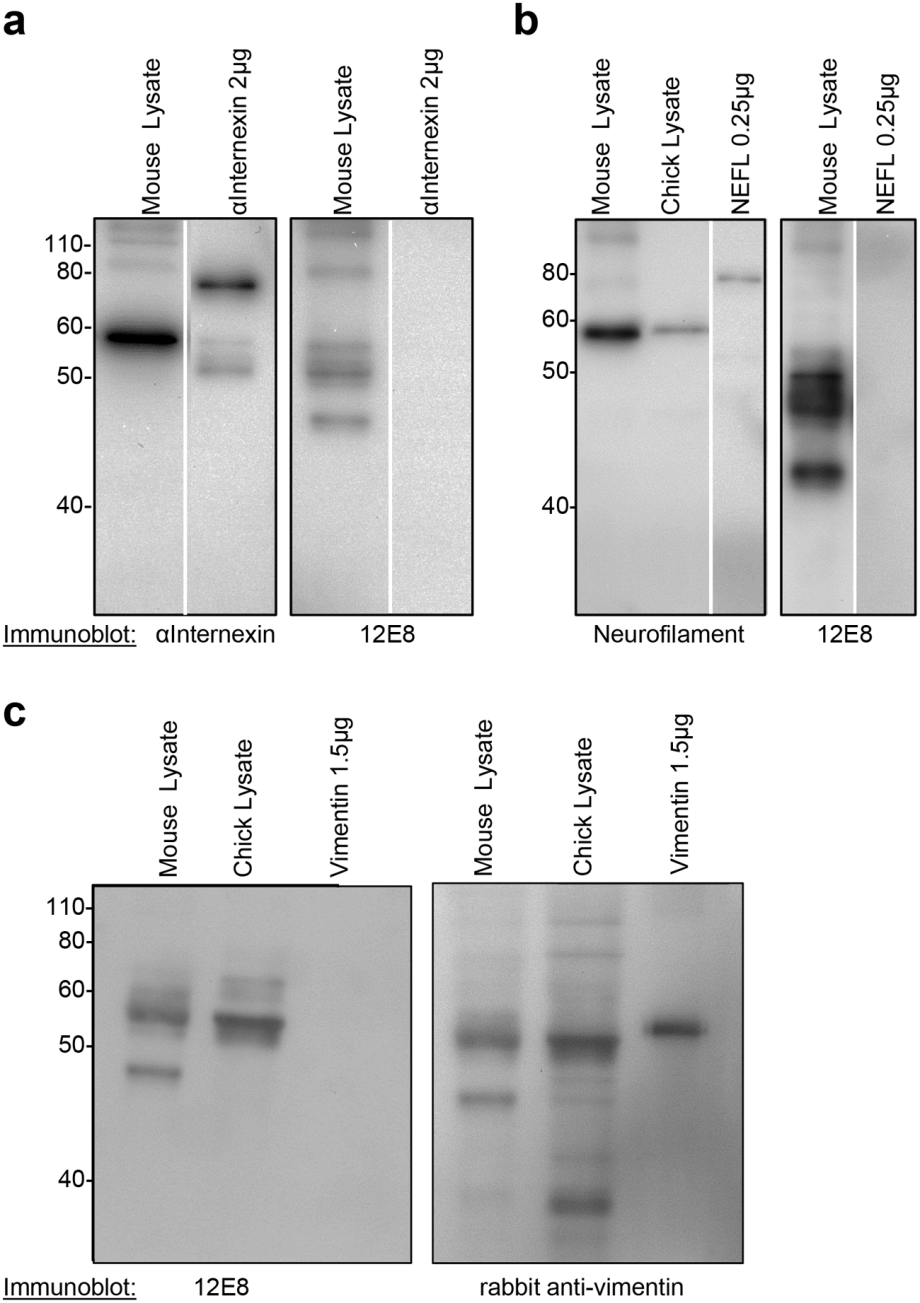
**No interaction between 12E8 antibodies and recombinant intermediate filament proteins on immunoblot.** (A) Recombinant α-internexin protein is not detected by 12E8 antibodies on immunoblot. (B) Recombinant light chain neurofilament protein is not detected by 12E8 on immunoblot. (C) Recombinant vimentin protein is not detected by 12E8 antibodies on immunoblot.

**Fig. 5.**
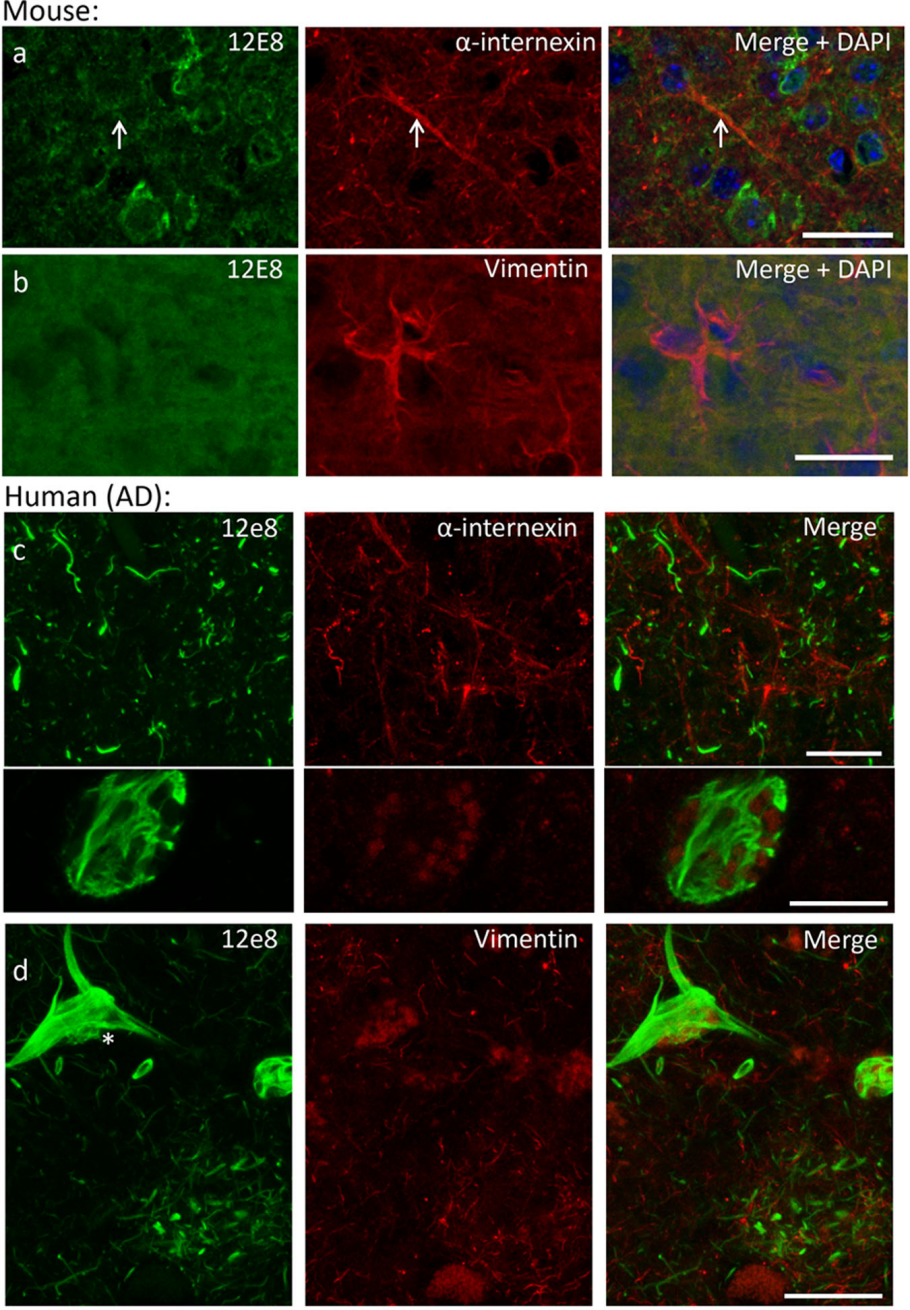
**No co-localisation of 12E8 and intermediate filament labelling by immunofluorescence in mouse or human brain sections.** Localisation of 12E8 and intermediate filament antibody labels in mouse and human brain. (A) 12E8 and α-internexin labelling do not co-localise in mouse brain (arrows). (B) 12E8 and vimentin antibody labelling do not co-localise in mouse brain. (C) 12E8 and α-internexin antibody labelling of neuritic pathology in human AD brain does not co-localise. (D) 12E8 and vimentin antibody labelling in human AD brain does not co-localise (asterisk). Both α-internexin and vimentin labelling was seen in cell processes throughout the neuropil of AD brain, but these were independent from 12E8 (and tau) labelled neurites. Scale bars: 20 µm, except c, bottom: 10 µm.

Intermediate filament proteins were prominent in 12E8 antibody IPs from mouse brain lysates (Tables 1,2). To evaluate whether intermediate filament proteins bind directly to the 12E8 antibody, immunoblots were performed using purified recombinant proteins. Recombinant vimentin, α-internexin and neurofilament polypeptides did not interact directly with 12E8 on immunoblots (Fig. 4) (a very faint band was seen on one 12E8 immunoblot of recombinant vimentin protein, but this was not reproduced in repeat experiments). Immunofluorescent labelling of wildtype postnatal mouse brain sections showed 12E8 labelled neuronal cell bodies in neocortex and this did not overlap with α-internexin labelling in parenchymal cell processes (Fig. 5A). Vimentin antibody labelled astrocytes in mouse brain that did not co-label with 12E8 antibody (Fig. 5B). Human autopsy AD brain sections also did not show co-localisation of 12E8 labelling and intermediate filament proteins α-internexin or vimentin (Fig. 5C,D).

### The 12E8 antibody binding antigen on ADF/cofilin-actin rods

ADF/cofilin-actin rods form in neuronal processes as a result of actin cytoskeletal rearrangement in rodent and chick neurons exposed to stressors such as ATP depletion, excitotoxicity, amyloid oligomers, proinflammatory cytokines or ischemia (Maloney et al., 2005; Minamide et al., 2000; Whiteman et al., 2009, 2011; Won et al., 2018). Rods and aggregates of cofilin are also seen in autopsy human AD brain tissue (Minamide et al., 2000; Rahman et al., 2014). The 12E8 antibody label localises to ADF/cofilin-actin rods generated in cultured rodent, chick and human neurons, but the antigen recognised by 12E8 on rods remains unclear (Fig. 7) (Whiteman et al., 2009). Since the 12E8 antibody IPs revealed intermediate filaments and the EIF3 protein complex as major immunoprecipitating components, we wanted to determine if any of these components localised to ADF/cofilin-actin rods along with 12E8 antibody labelling in cultured neurons.

**Fig. 6.**
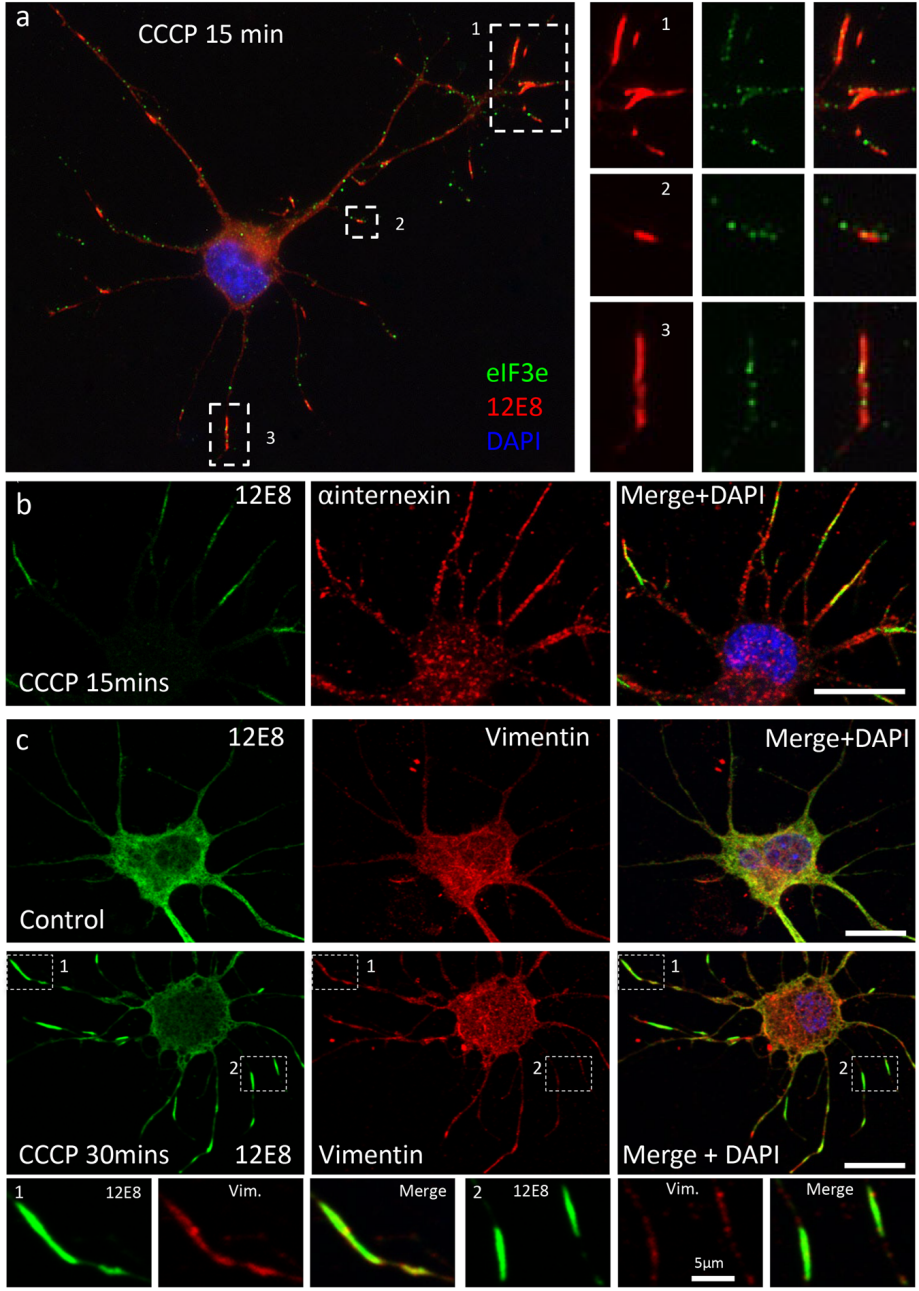
**Distribution of Eif3e and intermediate filament labelling relative to ADF/cofilin-actin rods in primary cultured chick neurons.** (A) EIF3e labelled puncta do not enrich in 12E8-labelled rods that are induced in primary cultured chick neurons by CCCP treatment. (B) α-internexin labelling shows some localisation to 12E8-labelled rods in CCCP-treated primary chick neurons. (C) Control and CCCP-treated primary chick neurons labelled with vimentin and 12E8 antibodies. Vimentin labelling localises in part to 12E8-labelled rods. Scale bars: 10 µm (inset C: 5 µm).

**Fig. 7.**
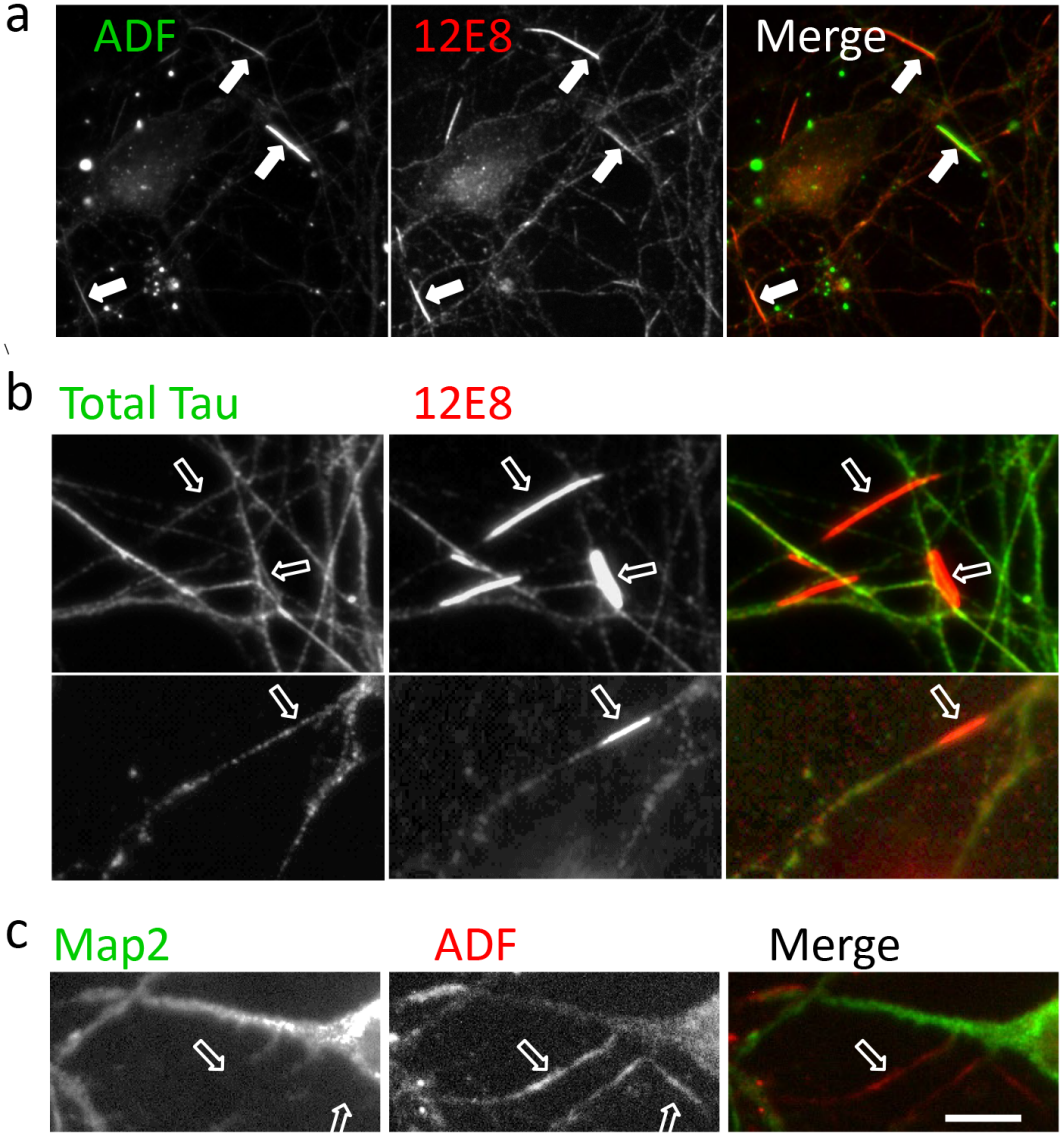
**Tau or MAP2 immunolabelling does not accumulate at ADF/cofilin-actin rods in primary cultured chick neurons: the antigen recognised by 12E8 on ADF/cofilin-actin remains uncertain.** (A) 12E8-labelled ADF/cofilin-actin rods generated in primary cultured chick neurons by CCCP-treatment (arrows). (B) Total tau protein immunolabel (rabbit polyclonal antibody, Dako), does not strongly enrich at 12E8-labelled rods (open arrows). (C) Mouse monoclonal HMW MAP2 antibodies do not co-label ADF/cofilin-actin rods in primary chick neurons. Scale bar: 5 µm.

ADF/cofilin-actin rods were generated in primary cultured chick neurons by ATP depletion using CCCP. EIF3e antibody labelled puncta that were relatively evenly distributed along neurites and did not enrich at ADF/cofilin-actin rods labelled with 12E8 antibodies (Fig. 6A). The α-internexin and vimentin antibody labelling did localise to 12E8-labelled rods to some extent (Fig. 6B,C, arrows). The 12E8 label on ADF/cofilin-actin rods (Fig. 7A) did not consistently co-localise with total tau (Dako) label (Fig. 7B) or with MAP2 labelling (Fig. 7C). Based on this data, we cannot narrow down the major contributing antigen recognised by the 12E8 antibody on ADF/cofilin-actin rods with certainty.

## DISCUSSION

The aim of this work was to identify proteins that bind directly or indirectly to the phospho-tau/MAP2 antibody 12E8, in neonatal mouse and embryonic chick brain. Known direct antigens of 12E8 – tau and MAP2 – were found in IPs from both mouse and chick brain lysates. The majority of other proteins detected mostly reflect indirect interactions with the 12E8 antibody (Table 1). The predominant proteins immunoprecipitated by 12E8 from mouse brains were MAP2, FSD1l, and intermediate filament proteins α-internexin, neurofilament and vimentin, with tau also detected in all IPs except for from tau null mouse brain lysates. The profile of proteins detected in the 12E8 IPs was different in chick brain and included MAP2, tau and subunits of the EIF3 protein complex. Intermediate filament proteins and FSD1l were not found in any of the chick brain IPs.

We used RIPA (radioimmunoprecipitation assay) buffer containing SDS and deoxycholate to disrupt protein-protein interactions because we wanted to mostly identify direct binding partners of 12E8, however, known tau/MAP2 binding partners, tubulin and actin were detected in most IPs from both mouse and chick brain, and the tau binding partner and kinase MARK4 was detected in mouse brain IP, highlighting that some relevant protein-protein complexes and consequently indirect interactions with the 12E8 antibody were maintained under the RIPA buffer conditions. We did not test other buffer conditions that might have maintained more protein-protein interactions or been less denaturing. FSD1l protein was a major component of the mouse brain lysate IPs, but whether this reflects a direct or indirect interaction with 12E8 in mouse remains unclear. FSD1l belongs to a family of microtubule-associated proteins that appear to be involved in the organisation of microtubules at the centrosome during cell division (Carim-Todd et al., 2001; Manabe et al., 2002; Stein et al., 2002). Little is known about the function of these proteins, however, there is evidence that like tau and MAP2, their association with microtubules is regulated by serine phosphorylation (Stein et al., 2002). This leaves open the possibility that FSD1l may interact with 12E8 directly through one of these phosphorylation sites, but this question requires further investigation. Overall, the results indicate that the interaction between the 12E8 antibody and intermediate filament proteins in mouse is indirect. The reason for the presence of intermediate filament protein subunits in the 12E8 IPs is unclear but might be explained by an indirect interaction between 12E8-binding tau or MAP2 and the common binding partner tubulin, as for example, neurofilament proteins have been shown to bind tubulin and to cross link microtubules (Miyasaka et al., 1993).

The identity of the 12E8- binding antigen on ADF/actin-cofilin rods remains unclear. In humans, ADF/cofilin-actin rods are associated with amyloid plaque pathology in autopsy AD brain tissue (Bamburg et al., 2010; Minamide et al., 2000). In embryonic chick or rodent animal models, similar rods are generated by reversible reorganisation of the actin cytoskeleton occurring during transient ATP depletion, that can be induced by treating the cultured neurons with mitochondrial transport chain inhibitors such as CCCP (rods can also be induced by many other stimuli; Bamburg et al., 2010; Minamide et al., 2000; Whiteman et al., 2009; Won et al., 2018). Rods formed *in vitro* contain actin and actin associated proteins ADF/cofilin and they also co-label with 12E8 antibodies (but not other phospho-tau antibodies) (Whiteman et al., 2011). The major protein components in chick brain IPs using 12E8 were tau, MAP2 and EIF3 complex proteins. We did not find evidence for EIF3e protein localisation in 12E8-antibody labelled rods generated in primary cultured chick neurons. Vimentin and neurofilament proteins were not found in 12E8 antibody IPs from chick brain, but they did partially localise to cofilin rods in the cultured chick neurons, although these intermediate filaments were also found along other stretches of neurite not labelled by 12E8 antibodies. Neither MAP2 (Won et al., 2018) or tau antibody labelling enriches in rods in rodent or chick neurons. We have been unable to narrow down the specific 12E8 antibody binding antigen that appears in ADF/cofilin-actin rods in these model systems.

In the mass spectrometry analysis, protein subunits of the EIF3 complex were found in 12E8 antibody IPs from embryonic chick brain, and EIF3a was in one IP from a tau null mouse brain lysate. IP and immunoblot further showed that 12E8 weakly immunoprecipitated Eif3e from mouse. We do not know the basis for the species difference, or why EIF3 proteins were more prominent in IPs from chick than mouse – it could potentially be due to developmental stage or other reasons. EIF3 is a large complex of 13 subunits that is involved in translation (Sha et al., 2009). Six of these subunits – predominantly EIF3e and EIF3a were co-immunoprecipitated by 12E8 antibodies in all three independent experiments using chick brain lysates. EIF3e has a MW (48 kDa) similar in size to bands detected by 12E8 on immunoblots from the same lysates (∼40-70 kDa), so we checked whether recombinant EIF3e protein directly binds to 12E8 and found that it does not. We did find that in addition to 12E8 antibodies, MAP2 or tubulin antibodies also co-immunoprecipitated EIF3e protein from chick brain. These data are consistent with a (direct or indirect) interaction between EIF3e and tubulin proteins, in line with previous studies that demonstrated an interaction between EIF3, as well as other components of the RNA translation apparatus, with microtubules or tubulin subunits (Hasek et al., 2000; Kozielski et al., 2011). The cytoskeleton is an organiser and regulator of translation (Baumann et al., 2022; Kim and Coulombe, 2010; Noma et al., 2017; Piper et al., 2015) and previous biochemical analysis has also suggested that EIF3 is involved in the assembly of ribosomal particles in the nucleolus (Sha et al., 2009). Our co-labelling data supports this, as we demonstrate that EIF3e prominently resides in the nucleolus in cultured chick neurons, in line with its proposed function there. The 12E8 antibody labelling in the same neurons was excluded from the nucleolus, suggesting that the EIF3e complexed with 12E8 antibody-interacting proteins detected in the IPs is not related to nucleolar functions. Indeed, double immunolabelling by the 12E8 antibody and EIF3e antibodies showed evidence of co-localisation in the cytosol but not in the nucleus or nucleolus. Co-localisation of immunolabelled phosphorylated tau (Ser396/404) and EIF3 complex proteins has been previously reported within stress granule (SG) aggregates of RNA protein complexes in htau transgenic mice (Vanderweyde et al., 2012). RNA SG particles are proposed to regulate energy consumption by shutting down non-essential translation during cell stress and may be associated with neurodegenerative disease (Kedersha et al., 2013). Tau is an RNA binding protein (Kampers et al., 1996), which might contribute to an association with other RNA binding proteins physiologically, in SGs, or in neurofibrillary inclusions (Brandt et al., 2020; Drummond et al., 2020; Sinsky et al., 2020, 2021; Vanderweyde et al., 2012). Further work is needed to determine whether interactions between phosphorylated tau/MAP2, RNA and EIF3 complex proteins are functionally relevant to RNA translation regulation or for mechanisms of neurodegenerative disease.

## MATERIALS AND METHODS

### Antibodies and proteins

Primary mouse monoclonal antibodies were: anti-phospho-tau/MAP2 (12E8, provided by Dr Peter Seubert, Prothena Biosciences); GAPDH (G8795, Sigma); tubulin (T6199, Sigma); MAP2 (M1460, Sigma); vimentin (ab20346, Abcam); neurofilament (ab72995, Abcam); α-internexin (mab5224, Chemicon); fibrillarin (ab4566, Abcam). Primary rabbit monoclonal antibodies were anti-tau (ab76128, ab64193, Abcam). Primary rabbit polyclonal antibodies were: anti-tau (Dako, Abcam); FUS (11570, Proteintech); α-internexin (ab5354, Chemicon); ADF/cofilin (D8815, Sigma); vimentin (PAB9580, Abnova); EIF3e (ab36766, Abcam). Secondary antibodies were anti-mouse or anti-rabbit HRP-conjugated (Jackson ImmunoResearch); Alexa fluor-conjugated 488, 555 and 647 goat anti-mouse and anti-rabbit (Invitrogen). Recombinant proteins were: EIF3e (ab114769, Abcam); α-internexin (ab160346, Abcam); neurofilament (H00004747, Abnova); vimentin (ab73843, Abcam).

### Lysate preparation

Tau null mice and tau null mice expressing htau, were obtained from Jackson Labs (Bar Harbor, ME, USA). Mouse and chick brain were dissociated in RIPA buffer (50 mM Tris-HCl, 1% triton-X-100, 2.5% sodium deoxycholate, 0.1% SDS, 10% NaCl,1 mM EGTA) containing Phos-Stop and Protease Inhibitors (Roche): - 500 µl per E8 (embryonic day 8) chick or E16 (embryonic day 16) mouse brain and 1000 µl per P0 mouse brain. The tissue was mechanically lysed using 22G sterile hypodermic needles and 1 mL syringes through pipetting action. The lysates were incubated on ice for 10 min, with frequent use of vortex during the incubation period to completely lyse the tissue. The sample was then centrifuged at 16,000 ***g***/4°C for 30 min and the supernatant was aliquoted and stored at −80°C until required.

### Immunoprecipitation and SDS-PAGE

12E8 antibody binding to Protein G-conjugated Dynabeads (Invitrogen) was achieved through incubation with rotation of 30 µL dynabeads (supernatant removed) and 1 µl of 12E8 antibody (7.5 µg) in 200 µl antibody binding and washing buffer [1X PBS, 0.01% (v/v) tween] for 10 min at room temperature. The supernatant was removed, and the antibody-bead complex was washed in 200 µl of washing buffer. Brain lysate (100 µL) was added to the antibody-bead complex and incubated with rotation for 10 min at room temperature. The beads were then washed three times and eluted in 15 µl denaturing Laemmli sample buffer containing 10% (v/v) mercaptoethanol at 95°C for 10 min. Samples were loaded on 8-10% SDS-Polyacrylamide gels for electrophoresis. IP controls included processing without the antibody (lysate+beads only) and processing without the lysate (beads+antibody only). Gels used subsequently for mass-spectrometry were prepared using filtered (0.22 µm) buffers and reagents and clean glassware to reduce contamination with dust particles and keratins. For immunoblotting, proteins were transferred to nitrocellulose membranes, blocked with 5% skim milk in TBST, washed, incubated in primary antibody and antibody labelled bands detected with HRP-coupled secondary antibodies using an ECL Western blotting detection System (Amersham) on a ChemiDoc XRS (Bio-Rad).

### Gel staining and mass spectrometry

SDS-PAGE gels were fixed in 10% methanol (v/v) and 7% (v/v) acetic acid for 30 min to 1 h (not exceeding 1 h) and then stained in 100 ml of Sypro Ruby overnight, returned to the fixing solution for 15 min, washed and imaged using a FLA9000 scanner. Gels were post-stained overnight with Coomassie Blue. After destaining, the gel was imaged with a GS-800 scanner sealed in sterilised moist plastic sheets and stored at 4°C for later use for mass spectrometry. Each lane was cut into four pieces and the proteins within each piece were trypsinised and analysed by mass spectrometry. The gel slices were diced up, destained 2-4 times for 10 min each in 60% (v/v) 20 mM ammonium bicarbonate, 40% (v/v) acetonitrile, then rinsed twice in acetonitrile, dried with a vacuum centrifuge (Concentrator 5301, Eppendorf, Hamburg, Germany) and rehydrated in approx. 100 µl of 12 ng/µl sequencing grade porcine trypsin (Promega, Sydney, Australia). Excess trypsin was removed and 20 mM ammonium bicarbonate added to cover the gel pieces and samples were then incubated at 37°C overnight. Peptides were concentrated and desalted using C18 micro-columns (Millipore, Billerica, MA, USA) according to the manufacturer's protocol, then eluted in 5 µl of 70% (v/v) acetonitrile and 0.1% (v/v) formic acid into a low-bind 96-well plate (Eppendorf, Dusseldorf, Germany) and then diluted with 45 µl of 0.1% (v/v) formic acid. Peptides were desalted on a ZORBAX 300SB-C18 trap (5 μm, 5×0.3 mm, Agilent Technologies) and separated using a an in-house prepared fritted nano C18 column packed with Reprosil–Pur (3 µm, 75 µm×150 mm; Dr Maish GmbH, Ammerbuch, Germany) on an Agilent 1100 HPLC system coupled to an AB Sciex QSTAR Elite Q-TOF MS for analysis. Peptides were eluted using a gradient of 5-40% buffer B mixed with buffer A [buffer A: 0.1% (v/v) formic acid; buffer B: 0.1% (v/v) formic acid in acetonitrile] at a flow rate of 0.3 µl/min. MS survey scans were performed over a range of 350-1750 m/z followed by three data-dependent MS/MS scans over a range of 65-2000 m/z. Two technical replicates were generated for each gel slice. Peak lists were generated using Analyst QS vr 2.0 (AB Sciex) and analysed using MASCOT version 2.4 (Matrix Science, London, UK) against *Homo sapiens*, *Mus musculus* and *Gallus gallus* specific databases. One missed cleavage per peptide, a mass tolerance of 0.2 Da (MS and MS/MS), and variable modification by oxidation of methionine were allowed in the MASCOT search. The MASCOT results were further analysed in Scaffold vr 3.5 (Proteome Software Inc, Portland, OR, USA).

### Cell culture and immunofluorescence labelling

Primary chick tectal neurons were prepared from freshly dissected chicken embryos (E8), as previously described and cultured for 2 to 7 days *in vitro* (div) on poly-D-lysine-glass coverslips in neurobasal media containing B27, L-glutamine and antibiotic/antimycotic (Invitrogen) (Whiteman et al., 2009). To generate ADF/cofilin-actin rods, cell cultures were ATP depleted by treatment in 5μM CCCP (carbonyl cyanide m-chlorophenyl hydrazone) for 15 min at 37°C. Cells were fixed with 4% paraformaldehyde at 37°C for 35 min, permeabilised for 90 s with 0.05% Triton X-100 in PBS, blocked in 5% goat serum and immunolabelled using primary antibodies and Alexa fluor-conjugated secondary antibodies with DAPI counter staining.

Human autopsy AD brain tissue use was approved by the University of Sydney Human Ethics Committee (protocol numbers 14432 and 8616). Sections (20-45 μm) of formalin-fixed human inferior temporal cortex (ITC) and anterior cingulate cortex (ACC) were obtained from the NSW Brain Bank network (cases are described in Davies et al., 2017). Free floating 45 μm postnatal day 0 (P0) wild-type mouse brain sections were used for immunolabelling. Sections were incubated for 2-3 h at RT or overnight at 4°C in primary antibodies. Bound antibody was visualised using Alexa fluor-conjugated secondary antibodies with Hoechst counter staining. For double labelling antibody binding was visualised using anti-Rabbit Alexa fluor 647 and anti-Mouse Alexa Fluor 555. Sections were mounted on slides (Menzer-Glass Ultra Plus) with Prolong Gold anti-fade (Invitrogen, P36930) for confocal microscopy imaging. After imaging of cell cultures or human tissue sections (see below), co-localisation analysis was performed using the ‘Just Another Colocalisation Programme’ (JACoP) plugin of ImageJ software with despeckling to first reduce background noise.

### Fluorescence microscopy

Primary cultured cells were imaged using an epifluorescence Zeiss Axio Observer inverted microscope containing a xenon light source, 40×NA1.4 Plan-Apochromat oil objective, and CCD camera driven by AxioVision software (4.8.2) with discrete filter sets segregating Alexa-fluor- 488, 555, 647 and DAPI/Hoechst channels. Human and mouse brain tissue sections were imaged using a Zeiss LSM 710 confocal microscope equipped with NA1.4 Plan-APOchromat oil immersion objectives; 488 nm, 543 nm, 633 nm and 405 nm lasers; and with pinholes maintained at 1 Airy Unit for each channel. Single labelling was performed to confirm separation of emissions in all multi-labelled samples.

## Supplementary Material

10.1242/biolopen.060067_sup1 Supplementary information
