# Supplementary material for: Protein complexes from mouse and chick brain that interact with phospho-KXGS motif tau/microtubule associated protein antibody
Source: Biol Open. 2024 Feb 27;13(2):bio060067. doi: 10.1242/bio.060067 (PMC10924212; doi:10.1242/bio.060067)
Supplement: Supplementary information [file biolopen-13-060067-s1.pdf]

Table S1.

a. Detailed list of peptides detected by mass spectrometry of 12E8 immunoprecipitates from P0 tau KO mouse brain

Protein	Accession	Peptides	m/z (charge)	Ion score	Identity Score
**Alpha internexin**	NP_666212.3				
		(R)DGLAEEVQR(L)	508.75(2)	71.01	38.29
		(R)EEIHEYR(R)	488.23(2)	66.7	38.0
		(R)EYQDLLNVK(M)	561.29(2)	61.2	37.4
		(R)FSTGGLSISGLNPLPNPSYLLPPR(I)	833.11(3)	119.3	33.5
			1248.16(2)	83.9	33.5
		(R)KLLEGEETR(F)	537.79(2)	63.8	37.7
		(K)LLEGEETR(F)	473.75(2)	54.6	38.1
		(R)LPASDGLDLSQAAAR(T)	742.88(2)	119.2	36.2
		(R)LSGPGGSGSFR(S)	511.25(2)	77.9	37.4
		(K)mALDIEIAAYR(K)	641.32(2)	83.7	36.4
		(R)QILELEER(H)	515.28(2)	54.7	37.8
		(R)RLPASDGLDLSQAAAR(T)	547.62(3)	125.8	36.1
		(R)TIEIEGLR(G)	465.76(2)	54.8	37.7
		(K)VESLLDELAFVR(Q)	695.87	105.8	36.7
		(K)VSSAGLSLK(K)	431.25	58.4	38.4
		(-)ALEAELAALR(-)	528.80(2)	88.1	37.3
		(-)AQLEEASSAR(-)	531.26(2)	77.2	37.3
		(-)AQYESLAAK(-)	490.75	63.2	37.2
		(-)DVDGATLAR(-)	459.23(2)	61.7	38.2
		(-)FANLNEQAAR(-)	567.29(2)	68.6	37.2
		(R)HSAEVAGYQDSIGQLESDLR(N)	725.67(3)	154.3	34.0
		(K)KVESLLDELAFVR(Q)	506.95(3)	86.3	36.4
		(-)NLQSAEEWYK(-)	634.30(2)	41.5	36.3
		(R)TNEKEQLQGLNDR(F)	515.59(3)	107.3	36.1
		(K)VGEGFEETLGEAVISTK(K)	883.43	115.2	35.5
		(-)VGELFQR(-)	424.73(2)	51.2	38.4
**Neurofilament light polypeptide**	P08551				
		(R)ALYEQEIR(D)	511.26(2)	53.2	37.4
		(R)FTVLTESAAK(N)	533.78(2)	62.5	37.1
		(R)IDSLmDEIAFLK(K)	705.85(2)	97.7	36.1
		(R)LAAEDATNEK(Q)	531.25(2)	57.5	37.3
		(R)LSFTSVGSITSGYSQSSQVFGR(S)	1148.05(2)	101.8	37.7
		(R)SAYSGLQSSSYLmSAR(S)	862.39(2)	122.8	35.6
		(R)SFPAYYTSHVQEEQTEVEETIEATK(A)	972.77(3)	140.1	31.4
		(K)VHEEEIAELQAQIQYAQISVEmDVSSKPDLSAALK(D)	972.22	111.5	29.9
		(K)VLEAELLVLR(Q)	577.85	96.7	36.3
		(-)EGLEETLR(-)	473.74(2)	73.9	38.1
		(K)QNADISAmQDTINK(L)	782.86(2)	115.1	35.7
		(R)SAYSSYSAPVSSSLSVR(R)	874.41(2)	98.3	35.2
		(R)SYSSSSGSLmPSLENLDLSQVAAISNDLK(S)	1010.47(3)	120.7	31.7
**Neurofilament medium polypeptide**	NP_032717.2				
		(K)AQVQLDSDHLEEDIHR(L)	476.98(4)	99.2	35.2
		(-)AVEEVITISK(-)	544.81(2)	80.8	37.5
		(-)EEIAEYR(-)	455.22(2)	58.2	36.9
		(-)EIEAEIQALR(-)	586.31(2)	66.9	37.1
		(-)GSPSTVSSSYK(-)	550.26(2)	85.2	36.9
		(K)QASHAQLGDAYDQEIR(E)	601.28(3)	121.7	35.7
		(-)SNEKEQLQGLNDR(-)	510.92(3)	74.9	36.0
		(R)SNHEEEVADLLAQIQASHITVER(K)	863.75(3)	139.1	33.3
		(-)VSGSPSSGFR(-)	490.74(2)	61.6	37.2
		(R)FSTFSGSITGPLYTHR(Q)	590.96(3)	142.2	35.7
		(K)FVEEIIEETKVEDEK(S)	612.97(3)	95.7	35.2
		(R)HNHDLSSYQDTIQQLENELR(G)	610.78(4)	71.5	33.5
		(R)QLSDIEER(H)	495.24(2)	70.3	37.8
		(K)SEmEETLTAIAEELAASAK(E)	670.65(3)	75.6	34.4
		(K)SIELESVR(G)	466.75(2)	52.1	38.5
		(K)VQSLQDEVAFLR(S)	702.87(2)	106.2	36.4
**FSD 1-like**	NP_001182213.1				

Biology Open • Supplementary information

**protein isoform**					
		(R)ALNFSLDNSSSHLNLK(V)	587.30(3)	76.4	35.3
		(K)AVAGDYSDPVTLETR(A)	797.38(2)	122.6	35.9
		(K)GTEYTLSGLK(F)	534.77(2)	70.0	37.1
		(R)SLDIKEPEEFSK(A)	474.57(3)	76.0	36.0
		(K)SYSVGVAYK(T)	487.25(2)	55.4	37.7
		(K)VSDNmTHLmVDFSQER(Q)	647.61(3)	86.1	33.6
**Vimentin**	NP_035831.2				
		(R)EEAESTLQSFR(Q)	648.8(2)	55.3	36.7
		(K)LHDEEIQELQAQIQEQHVQIDVDVSKPDLTAALR(D)	977.99(4)	72.8	29.4
		(R)QDVDNASLAR(L)	544.76(2)	109.8	37.6
		(R)QVDQLTNDK(A)	530.76(2)	55.3	37.9
		(R)SLYSSSPGGAYVTR(S)	722.85(2)	63.7	36.4
**Alpha Tubulin**	NP_033473.1				
		QLFHPEQLITGK	470.92(3)	85.2	36.1
		(K)TIGGGDDSFNTFFSETGAGK(H)	1004.44(2)	121.2	33.8
**Beta Tubulin**	NP_035785.1				
		(R)ALTVPELTQQVFDAK(N)	830.43(2)	90.58	36.520
		(K)mAVTFIGNSTAIQELFK(R)	943.47(2)	94.0	35.3
**Microtubule associated protein 2 (Map 2)**	P20357				
		(-)FAAPAQPEEER(-)	622.80(2)	60.0	36.9
		(K)TGVIQTSTEQSFSK(E)	756.87(2)	79.6	36.1
		(K)VELFGLGITYDQASTK(E)	871.44(2)	97.7	35.8
		(R)DLATDLSLIEVK(L)	658.86(2)	39.5	36.9
		(R)GSAQESLDTISPK(N)	666.82(2)	76.0	36.9
		(R)LASVSADAEVAR(R)	594.80(2)	75.3	37.2
		(R)SILTEQLETIPK(E)	686.38(2)	65.9	36.3
**Cytoplasmic Actin**	NP_031419.1*				
		(K)AGFAGDDAPR(A)	488.73(2)	72.7	37.6
		(R)AVFPSIVGR(P)	473.28(2)	42.1	38.1
		(K)SYELPDGQVITIGNER(F)	895.93(2)	92.5	35.7
		(R)VAPEEHPVLLTEAPLNPK(S)	652.02(3)	104.5	34.9
**Eukaryotic translation initiation factor 3 subunit A (EIF3A)**	NP_034253.3				
		(R)FSVLQYVVPEVK(D)	704.38(2)	71.0	36.5
		(R)ITTmQLER(E)	504.25(2)	37.9	37.3
		(R)LTSLVPFVDAFQLER(A)	867.96(2)	47.5	36.0
		(R)NQLTAmSSVLAK(A)	639.83(2)	101.4	36.6
		(-)IGLINDmVR(-)	523.78(2)	50.6	37.7
		(-)LLDmDGIIVEK(-)	631.33(2)	70.3	36.9
		(-)VLLATLSIPITPER(-)	761.96(2)	48.3	35.2

Biology Open • Supplementary information

b. Detailed list of peptides detected by mass spectrometry of 12E8 immunoprecipitates from P0 tau KO-htau transgenic mouse brain

Protein	Accession	Peptide	Ion score	Identity Score	m/z (charge)
**Alpha internexin**	NP_666212.3				
		(R)ALEAELAALR(Q)	79.0	37.3	528.80(2)
		(-)AQALLER(-)	41.4	38.0	400.73(2)
		(R)AQLEEASSAR(A)	52.5	37.3	531.26(2)
		(R)DGLAEEVQR(L)	63.7	38.3	508.72(2)
		(-)DVDGATLAR(-)	39.5	38.2	459.23(2)
		(-)EQLQGLNDR(-)	55.1	38.5	536.77(2)
		(-)FAVFIEK(-)	44.7	36.4	427.24(2)
		(-)FSTGGLSISGLNPLPNPSYLLPPR(-)	81.8	33.5	833.11(3)
		(R)HSAEVAGYQDSIGQLESDLR(N)	167.3	34.3	725.67(2)
		(K)KVESLLDELAFVR(Q)	87.8	36.4	506.95(3)
		(-)LLEGEETR(-)	53.5	38.1	473.74(2)
		(K)mALDIEIAAYR(K)	90.3	36.4	641.32(2)
		(-)VGEGFEETLGEAVISTK(-)	160.6	35.5	883.43(2)
		(-)VGELFQR(-)	54.4	38.4	424.73(2)
		(-)VSSAGLSLK(-)	49.5	38.4	431.25(2)
		(R)AQYESLAAK(N)	70.3	37.2	490.75(2)
		(R)EEIHEYR(R)	48.1	38.0	488.23(2)
		(R)EYQDLLNVK(M)	60.4	37.4	561.29(2)
		(R)KLLEGEETR(F)	56.9	37.7	537.78(2)
		(R)LPASDGLDLSQAAAR(T)	113.6	36.2	742.88(2)
		(R)QVHDEEVAELLATLQASSQAAAEVDVAVAKP-DLTSALR(E)	139.5<br>32.6	29.5<br>29.2	987.25(4)<br>1315.99(3)
		(R)RLPASDGLDLSQAAAR(T)	130.2	36.1	547.62(3)
		(R)TIEIEGLR(G)	48.1	37.7	465.76(2)
		(R)TNEKEQLQGLNDR(F)	89.9	36.1	515.59(3)
		(K)VESLLDELAFVR(Q)	103.4	36.7	695.87(2)
		(R)FSTFSGSITGPLYTHR(Q)	135.1	35.7	590.96(3)
		(K)TDISTALK(E)	46.4	38.4	424.74(2)
		(R)VSGSPSSGFR(S)	70.2	37.2	490.74(2)
**Neurofilament medium polypeptide**	NP_032717.2				
		(K)AQVQLDSDHLEEDIHR(L)	107.6	35.1	635.63(3)
		(-)AVEEVITISK(-)	84.4	37.5	544.81(2)
		(-)EIEAEIQALR(-)	85.1	37.1	856.31(2)
		(-)FAGYIEK(-)	37.4	36.9	414.22(2)
		(-)FVEEIIEETK(-)	40.0	36.7	618.81(2)
		(R)GSPSTVSSSYK(R)	80.6	36.9	550.26(2)
		(R)LRDDTEAAIR(A)	58.2	37.3	580.3(2)
		(K)QASHAQLGDAYDQEIR(E)	87.4	35.6	601.28(3)
		(-)QLSDIEER(-)	46.9	37.8	495.24(2)
		(-)SIELESVR(-)	52.2	38.5	466.75(2)
		(R)SNEKEQLQGLNDR(F)	44.2	36.0	510.92(3)
		(R)SNHEEEVADLLAQIQASHITVER(K)	178.0	33.3	863.75(3)
		(K)VQSLQDEVAFLR(S)	101.5	36.4	702.87(2)
		(K)VHYLEQQNK(E)	28.3	37.3	579.79(2)
**Neurofilament light polypeptide**	P08551				
		(R)ALYEQEIR(D)	50.8	37.4	511.26(2)
		(R)EGLEETLR(N)	73.7	38.1	473.74(2)
		(R)IDSLmDEIAFLK(K)	97.7	36.1	705.85(2
		(R)LAAEDATNEK(Q)	79.1	37.3	531.25(2)
		(R)LSFTSVGSITSGYSQSSQVFGR(S)	124.5	37.7	1148.04(2)
		(R)SAYSSYSAPVSSSLSVR(R)	41.4	35.2	874.41(2)
		(K)VHEEEIAELQAQIQYAQISVEmDVSSKPDLSAALK(D)	70.3	29.9	972.22(4)
		(K)VLEAELLVLR(Q)	87.8	36.3	577.85(2)
		(R)YEEEVLSR(E)	53.2	37.1	512.75(2)
		(R)FSTFSGSITGPLYTHR(Q)	135.1	35.7	590.96(3)
		(K)TDISTALK(E)	46.4	38.4	424.74(2)
		(R)VSGSPSSGFR(S)	70.2	37.2	490.74(2)
		(R)FTVLTESAAK(N)	61.6	37.1	533.78(2)
		(-)KGADEAALAR(-)	47.0	38.0	501.27(2)
		(K)QNADISAmQDTINK(L)	92.3	35.8	782.86(2)
		(R)SAYSGLQSSSYLmSAR(A)	131.8	35.6	862.39(2)
		(R)SYSSSSGSLmPSLENLDLSQVAAISNDLK(S)	101.3	31.7	1010.47(3)

Biology Open • Supplementary information

**Microtubule associated protein tau**	NP_058525.1				
		(R)HLSNVSSTGSIDmVDSPQLATLADEVSASLAK(Q)	235.5	31.7	1087.18(3)
		(K)IGSLDNITHVPGGGNK(K)	94.9	36.0	526.94(3)
		(R)LQTAPVPmPDLK(N)	74.2	37.1	663.35(2)
		(K)TDHGAEIVYK(S)	51.1	37.4	566.78(2)
		(K)VQIVYKPVDLSK(V)	67.7	36.8	463.60(3)
		(-)IGSTENLK(-)	45.4	38.4	431.23(2)
		(-)TPSLPTPPTR(-)	58.1	37.1	533.79(2)
**Microtubule associated protein 2 (Map2)**	P20357				
		(K)GGQVQIVTK(K)	72.4	37.9	465.27(2)
		(R)IVQVVTAEAVAVLK(G)	113.6	35.8	720.43(2)
		(R)LINQPLPDLK(N)	59.1	37.3	575.83(2)
		(K)SGILVPSEK(K)	50.5	37.9	456.26(2)
		(R)DLATDLSLIEVK(L)	88.6	36.9	658.86(2)
		(-)FAAPAQPEEER(-)	56.1	36.9	622.79(2)
		(-)GSAQESLDTISPK(-)	67.6	36.9	666.83(2)
		(R)LASVSADAEVAR(R)	91.4	37.2	594.81(2)
		(R)SILTEQLETIPK(E)	74.3	36.3	686.38(2)
		(-)TGVIQTSTEQSFSK(-)	92.2	36.1	756.87(2)
		(R)TPGTPGTPSYPR(T)	62.8	37.1	615.80(2)
		(-)VSDFGQmASGmNVDAGK(-)	41.2	34.6	873.36(2)
**FSD-like protein isoform**	NP_001182213				
		(K)ALDVPVPEK(I)	58.7	36.6	484.27(2)
		(K)ALNFSLDNSSSHLNLK(V)	104.1	35.3	587.3(3)
		(-)AVAGDYSDPVTLETR(-)	106.1	35.9	797.38(2)
		(K)GTEYTLSGLK(F)	67.2	37.1	534.78(2)
		(-)SDEIQNFIDTLNHTLK(-)	97.8	35.4	629.98(3)
		(R)SLDIKEPEEFSK(A)	76.0	36.0	474.57(3)
		(-)SYSVGVAYK(-)	56.4	37.7	487.25(2)
		(K)VSDNmTHLmVDFSQER(Q)	136.2	33.5	647.61(3)
		(-)VTmASAFR(-)	50.6	37.1	449.72(2)
**Vimentin**	NP_035831	(K)FADLSEAANR(N)	54.2	37.6	547.26(2)
		(K)ILLAELEQLK(G)	63.3	36.5	585.35(2)
		(-)ISLPLPTFSSLNLR(-)	61.3	63.0	779.44(2)
		(R)QDVDNASLAR(L)	79.6	37.6	544.76(2)
**MAP /microtubule affinity-regulating kinase 4**	NP_758483				
		(K)IADFGFSNEFTLGSK(L)	81.0	36.1	816.88(2)
		(K)LFEVIETEK(T)	43.2	37.0	554.29(2)
**Myosin 10**	NP_780469	(R)ALELDPNLYR(I) \	56.1	37.2	602.31(2)
		(K)VVSSVLQFGNISFK(K)	91.5	36.0	762.91(2)
**heat shock cognate 71 kDa protein (HSC70)**	NP_112442				
		(R)ARFEELNADLFR(G)	71.7	35.9	494.25(3)
		(K)DAGTIAGLNVLR(I)	90.8	37.3	600.33(2)
		(R)FEELNADLFR(G)	80.4	36.4	627.30(2)
		(R)IINEPTAAAIAYGLDK(K)	108.7	36.5	830.43(2)
		(K)mKEIAEAYLGK(T)	65.9	36.6	423.55(3)
		(K)QTQTFTTYSDNQPGVLIQVYEGER(A)	121.8	32.6	925.43(3)
		(K)SENVQDLLLLDVTPLSLGIETAGGVmTVLIK(R)	138.7	30.8	1,085.57(3)
		(K)SINPDEAVAYGAAVQAAILSGDK(S)	106.1	34.4	754.04(3)
		(K)SQIHDIVLVGGSTR(I)	52.4	36.3	494.60(3)
		(K)STAGDTHLGGEDFDNR(M)	109.9	34.9	564.57(3)
		(K)TVTNAVVTVPAYFNDSQR(Q)	128.8	34.5	661.32(3)

Biology Open • Supplementary information

c. Detailed list of peptides detected by mass spectrometry of 12E8 immunoprecipitates from WT E16 mouse brain

Protein	Accession	Peptides	Ion score	Identity score	m/z (charge)
**Alpha-internexin**	NP_666212.3				
		(R)ALEAELAALR(Q)	75.85	37.29	528.81(2)
		(R)EYQDLLNVK(M)	61.45	37.39	561.29(2)
		(K)FANLNEQAAR(S)	62.62	37.20	567.29(2)
		(R)HSAEVAGYQDSIGQLESDLR(N)	118.15	34.09	725.68(3)
		(K)KVESLLDELAFVR(Q)	79.67	35.78	506.97(3)
		(R)LSGPGGSGSFR(S)	68.43	37.40	511.26(2)
		(R)QILELEER(H)	51.08	37.79	515.28(2)
		(R)QVHDEEVAELLATLQASSQAAAEVDVAVAKPDLTSALR(E)	129.77	29.15	987.25(4)
		(R)TIEIEGLR(G)	57.4	37.73	465.77(2)
		(R)TNEKEQLQGLNDR(F)	60.18	36.09	515.61(3)
		(R)VGELFQR(E)	51.05	38.37	424.75(2)
		(R)FSTGGLSISGLNPLPNPSYLLPPR(I)	87.95	33.34	833.11(3)
		(R)LPASDGLDLSQAAAR(T)	114.9	36.21	742.90(2)
		(K)MALDIEIAAYR(K)	83.54	36.43	641.32(2)
		(K)VGEGFEETLGEAVISTK(K)	137.85	35.56	883.46(2)
**Microtubule associated protein Tau**	NP_034968.3				
		(K)AEEAGIGDTPNQEDQAAGHVTQAR(V)	163.76	33.0	822.37(3)
		(K)IGSLDNITHVPGGGNK(K)	82.73	35.92	526.96(3)
		(R)LQTAPVPmPDLK(N)	61.11	37.10	663.35(2)
		(R)TPSLPTPPTR(E)	57.56	37.13	533.79(2)
		(K)VQIVYKPVDLSK(V)	71.99	36.03	463.62(3)
**Beta tubulin**	NP_076205.1	(R)ALTVPELTQQmFDSK(N)	72.41	35.80	862.42(2)
		(R)INVYYNEATGNK(Y)	65.61	36.50	693.33(2)
		(R)YLTVAAIFR(G)	66.77	36.49	527.32(2)
**Vimentin**	NP_035831.2	(K)FADLSEAANR(N)	78.59	37.56	547.27(2)
		(K)ILLAELEQLK(G)	62.4	36.43	585.36(2)

Biology Open • Supplementary information

d. Detailed list of peptides detected by mass spectrometry of 12E8 immunoprecipitates from E7 chick brain

Protein	Accession	Peptides	m/z (charge)	Ion score	Identity score
**Microtubule associated protein tau**	NP_001186122.1				
		(K)IGSLDNISHVPGGGNK(K)	522.21(3)	49.6	28.81
			522.25(3)	48.3	29.96
		(R)QHVPSAEEAGIGATPSLEDHAAGDAAQAR(I)	714.80(4)	61.8	22.64
			714.82(4)	79.6	25.2
			714.79(4)	95.6	25.0
		(-)VQPSAAPmPDLK(-)	635.25(2)	43.7	29.59
			635.32(2)	53.4	30.91
			635.28(2)	51.0	39.1
		(-)TPSLPTPPAR(-)	518.73(2)	46.1	31.35
		(R)LSNVSSTGSINmVDSPQLATLADEVSASLAK(Q)	1,041.18(3)	127.8	26.06
		(K)VQIVYKPVDLSHVTSK(C)	604.99(3)	56.2	29.0
			604.96(3)	78.5	28.3
**Microtubule associated protein 2 (Map 2)**	XP_421857				
		(K)VGSLENAHHVPGGGNVK(I)	557.90(3)	48.44	28.23
		(-)VDHGAEIITQSPGR(-)	493.87(3)	43.58	28.49
		(R)TPGTPGTPSYSR(T)	610.72(2)	60.46	29.22
		(R)VINQPLPDLK(N)	568.77(2)	39.83	30.98
**Eukaryotic translation initiation factor 3 subunit E**	NP_001006349.1				
		(R)HLVFPLLEFLSVK(E)	771.36(2)	54.6	30.0
			771.44(2)	60.2	29.6
		(-)mLFDYLAEK(-)	573.21(2)	61.8	29.4
			573.28(2)	50.2	31.6
		(K)NLYSDEIPHALR(E)	476.53(3)	46.5	29.3
			476.58(3)	47.8	30.6
		(-)YLTTAVITNK(-)	562.25(2)	58.7	30.8
			562.30(2)	59.2	30.9
		(-)NALSSLWGK(-)	488.25(2)	46.8	32.1
		(K)LDLLSDTNmVDFAmDVYK(N)	1061.48(2)	107.2	28.1
		(K)LGHVVmGNNAVSPYQQVIEK(T)	733.71(3)	71.1	28.1
		(K)NLYSDEIPHALR(E)	476.57(3)	47.8	30.6
		(R)QEYLDTLYR(Y)	600.78(2)	65.4	31.2
		(K)QLQAETEPIVK(M)	628.33(2)	68.2	31.2
**Eukaryotic translation initiation factor 3 subunit F**	XP_421624				
		(K)AYVSAPmGVPGK(T)	596.73(2)	45.7	30.4
			596.80(2)	46.6	31.20
		(R)LHPVVLASIVDSFER(R)	561.25(3)	89.7	29.2
			561.29(3)	69.2	29.63
		(-)FLmDLINQVPK(-)	667.28(2)	56.2	30.37
			667.35(2)	54.1	30.73
		(-)VIGTLLGTVDK(-)	558.27(2)	91.9	31.38
		(R)IQDTLGmVLQYAEDVLSGK(V)	699.34(3)	100.1	28.65
		(R)VIGLSSDLQQVGSASAR(I)	844.44(2)	134.3	29.94
**Eukaryotic translation initiation factor 3 subunit A**	XP_421787				
		(R)FNVVQY VVPEVK(E)	710.89(2)	63.3	30.6
		(R)LLDmDGIIVEK(Q)	631.31(2)	65.4	31.4
		(R)LLQQVAQIYQSIEFSR(L)	641.66(3)	78.8	29.07
		(R)VLLATLSIPITPER(T)	761.95(2)	78.1	29.43
		(-)FNVVQYVVPEVK(-)	710.87(2)	71.9	30.6
		(-)NQLTAmSSALAK(-)	625.82(2)	74.0	31.03
		(R)SAPAPAAPPASAPPSASK(D)	787.91(2)	84.3	30.15
**Eukaryotic translation**	NP_001012553				

Biology Open • Supplementary information

**initiation factor 3 subunit L**		(K)LAGFLDLTEQEFR(I)	769.89(2)	89.4	30.22
		(K)mLGYFSLVGLLR(L)	692.88(2)	68.2	30.36
		(K)VFADEVQQQAQLSTIR(S)	916.97(2)	123.4	29.42
		(K)VYEIQDIYENSWTK(L)	894.42(2)	69.9	29.45
**Eukaryotic translation initiation factor 3 subunit H**	NP_001026122	(K)LFmAQALQDYNN(-)	722.33(2)	63.9	30.42
		(K)QVQIDGLVVLK(I)	606.34(2)	71.0	30.86
**Eukaryotic translation initiation factor 3 subunit B**	XP_003642190	(K)GTQGVVTNFEIFR(M)	734.39(2)	84.2	30.21
		(-)IINEFYPEADGK(-)	698.32(2)	72.82	30.27
**Cytoplasmic Actin**	XP_001236316	(-)EITALAPSTmK(-)	589.23(2)	46.3	31.52
			589.30(2)	46.8	32.05
		(R)GYSFTTTAER(E)	566.70(2)	58.2	29.12
			566.74(2)	62.1	31.55
		(K)QEYDESGPSIVHR(K)	506.22(3)	73.4	30.04
		(R)TTGIVmDSGDGVTHTVPIYEGYALPHAILR(L)	800.70(4)	62.9	22.69
		(K)DLYANTVLSGGTTmYPGIADR(M)	744.34(3)	69.2	28.0
		(K)SYELPDGQVITIGNER(F)	895.94(2)	86.2	29.7
		(R)VAPEEHPVLLTEAPLNPK(A)	652.01(3)	46.7	28.9
**Alpha-tubulin**	sp*P02552*				
		(-)AVFVDLEPTVIDEVR(-)	851.45(2)	80.39	29.71
			851.40(2)	67.5	29.0
		(-)DVNAAIATIK(-)	508.30(2)	40.43	32.10
			508.26(2)	59.0	31.4
		(-)EIIDLVLDR(-)	543.31(2)	71.87	30.58
			543.27(2)	77.4	30.8
		(R)LIGQIVSSITASLR(F)	729.43(2)	84.9	29.8
		(-)LIGQIVSSITASLR(-)	729.39(2)	57.0	29.6
		(-)NLDIERPTYTNLNR(-)	573.59(3)	66.3	28.9
**Beta-tubulin**	NP_990646				
		(R)AILVDLEPGTmDSVR(S)	816.42(2)	31.0	29.7
		(K)GHYTEGAELVDSVLDVVR(K)	653.63(3)	86.5	28.7
**PA28 proteasome activator complex subunit 3**	NP_001012568	(R)ITSEAEDLVANFFPK(K)	840.83(2)	99.1	27.3
		(R)NQYVTLHDmILK(N)	497.54(3)	28.3	29.1
		(K)SNQQLVDIIEK(V)	643.78(2)	68.9	30.29
		(R)TVESEAASYLDQISR(Y)	834.81(2)	115.2	25.4
		(K)YPHVEDYRR(T)	412.16(3)	28.81	30.02

Biology Open • Supplementary information
